# “That’s What the Program Is All about… Building Relationships”: Exploring Experiences in an Urban Offering of the Indigenous Youth Mentorship Program in Canada

**DOI:** 10.3390/ijerph18020733

**Published:** 2021-01-16

**Authors:** Leah J. Ferguson, Tammy Girolami, Reed Thorstad, Carol D. Rodgers, M. Louise Humbert

**Affiliations:** 1 College of Kinesiology, University of Saskatchewan, Saskatoon, SK S7N 5B2, Canada; reed.thorstad@usask.ca (R.T.); louise.humbert@usask.ca (M.L.H.); 2 Community Advisor, Saskatoon, SK, Canada; girolamit@spsd.sk.ca; 3 Faculty of Health Sciences, Ontario Tech University, Oshawa, ON L1G 0C5, Canada; Carol.Rodgers@ontariotechu.ca

**Keywords:** mentorship, Indigenous, school-based, communal-led, wellness, urban

## Abstract

Peer mentorship is an effective approach for delivering health promotion programs that may be particularly useful among underrepresented populations. Advancing the peer-led approach, the Indigenous Youth Mentorship Program (IYMP) is a communal-led program rooted in Indigenous values aimed at the promotion of healthy lifestyles in children and youth. The program includes layers of multi-age mentoring (i.e., elementary students, high school student mentors, and young adult health leaders [YAHLs]) and incorporates three core components: physical activity, healthy eating, and cultural teachings. The purpose of this study was to qualitatively explore elementary student, mentor, and YAHL experiences in an urban IYMP offering. Eleven sharing circles were conducted; six with elementary students (*n* = 23; grade 4 and 5 students), two with mentors (*n* = 3; students enrolled in a grade 10 wellness girls class), and three with YAHLs (*n* = 6; undergraduate university students). Focus groups were also held with respective school teachers and principals. An inductive content analysis generated three themes that represent the perceived impacts of this urban IYMP offering: (1) Fostering Wellness, (2) Strengthening Meaningful Connections, and (3) Exploring Leadership. Findings are positioned within a communal mentorship framework that is circular and multi-directional. By bringing together Indigenous and non-Indigenous peoples, this program offering supports Indigenous cultural relevance in an urban-based wellness program.

## 1. Introduction

Peer mentoring in health promotion programs is an effective method of delivering content and enhancing wellness, and it may be particularly useful among underrepresented populations [1]. Peer mentoring includes a high level of interaction among members of a similar age or status group, and thus the advantage of sharing health information in meaningful and relatable ways [2]. Effective health-promotion programs are paramount for Indigenous peoples in Canada who have higher rates of preventable chronic disease than non-Indigenous peoples [3] (The intentional use of the term Indigenous in this paper refers to the First Nations, Inuit, and Métis peoples in Canada [4]. The original inhabitants of Canada are distinct groups with unique languages, histories, and traditional practices.). Persisting inequities between Indigenous and non-Indigenous peoples in Canada, such as increased experiences of obesity, diabetes, cardiovascular disease, and mental health challenges, stem directly from long-term impacts of colonization including assimilation practices, cultural suppression, and historic trauma [5,6,7]. Indigenous youth, in particular, tend to have disproportionately high health problems due to a myriad of complex issues, including historical and contemporary colonization and intergenerational trauma [8]. To diminish the prevalence of health problems, health promotion professionals recommend focusing on prevention and creating environments that support a child’s development and positive health outcomes [9]. It is therefore important to center Indigenous youth voices and experiences [10,11] to best create programs that meet their needs and consider our colonial past and present. Creating environments where Indigenous youth are involved in program delivery has much merit for health and wellness.

Recognizing, honoring, and celebrating the strengths of Indigenous youth can have meaningful impacts on health and wellness. There is a small but growing body of scientific evidence that Indigenous peer-led programs benefit Indigenous children [12]. As children and youth spend a significant amount of their day at school, school-based health promotion programs offer another favorable strategy to improve health and wellness [1]. A recent systematic review found that Indigenous youth peer-led health promotion programming in Australia, Canada, New Zealand, and the United States led to improved health-related knowledge, attitudes, and behaviors [13]. The review included 20 interventions, seven of which were school-based, with many using cultural and artistic activities (e.g., dance, music, performances) as a means of engaging youth. The interventions varied in terms of structured classes (e.g., school curricula versus one-off information sessions), and many interventions incorporated Indigenous cultural education (e.g., medicine wheel, colonization). From their review, Vujcich et al., found that peer-led health promotion programs by Indigenous youth led to changes in healthy lifestyle knowledge, behaviors, and attitudes, including increased use of health services, decreased alcohol or substance use, increased awareness of sexual health issues, improved healthy lifestyle knowledge, and improved self-confidence and self-esteem. As less than ¼ of the studies included in the review took place in Canada, it is important to further explore the Canadian context in order to understand unique features and impacts of Indigenous youth peer-led health promotion programs in Canada.

Indigenous youth-focused programs in Canada have also included cultural and artistic activities to promote health and wellness. For example, an Indigenous youth-focused program in Behchokǫ, a First Nation community in the Northwest Territories, consisted of a five-day workshop where youth embraced a leadership role to direct creative art projects (e.g., music, visual arts, theatre [14]). As a result of the workshop, youth developed new skills, nurtured positive relationships, embraced their culture, and worked towards social change to promote healthy minds, bodies, and spirits through creative arts [14]. Another program, known as Healthy Buddies^TM^, created in British Columbia, was a school-based, peer-led program that included more traditional education or curricula [15]. Teachers worked with students in older grades to introduce and deliver concepts, and the older students were then paired with “buddies” from younger grades who received lessons on healthy eating, fitness, and body image. Children involved in the program experienced improved body mass index, healthy living, nutrition knowledge, self-esteem, and waist circumference. These studies suggest that Indigenous youth peer-led programs may be effective in promoting knowledge about, attitudes towards, and improved positive lifestyle behaviors. 

More research is needed to explore and understand Indigenous youth-focused health promotion programs in Canada. Among the almost 1.7 million people who self-identified as Indigenous in Canada in 2016, over 60% live in urban centers (i.e., a population of at least 1000 and a density of 400 or more people per square kilometer) [16]. With the majority of Indigenous peoples in Canada dwelling in small, medium, or large population centers, delivering and evaluating urban-based and youth-focused health promotion programs may provide insight into unique program benefits. 

The Indigenous Youth Mentorship Program (IYMP) is a youth-focused, communal-led program that originated in Winnipeg, Manitoba, Canada [17]. The IYMP grew out of the Rec and Read Mentorship Program for All Nations [18], which received the 2014 MacJannet Prize for community development and the 2015 Manitoba Mino Bimaadiziwin award for healthy living. The IYMP is unique from other Indigenous health promotion programs in that it integrates Indigenous values aimed at the promotion of healthy lifestyles in children and youth. The program is rooted in the Circle of Courage, which is a resilience-based theory intended to build on the strengths of youth as they assume leadership roles in their community [19], and the Four Rs—respect, relevance, reciprocity, and responsibility—which promote Indigenous ways of knowing, teaching, and learning [20]. The IYMP embraces a non-hierarchical, communal mentoring approach whereby learning is not just peer-led but goes in all directions. More specifically, the IYMP consists of interconnected layers of multi-age mentoring, whereby young adult health leaders (YAHLs) in the community support high school student mentors to design a program for elementary students. The mentors then deliver the program to the elementary students, with the YAHLs’ supervision and support. Each program session consists of three core components: (1) physical activity, (2) cultural teaching or activity, and (3) a healthy snack. The IYMP has been implemented in more than 20 communities across Canada, with each program offering taking a community-tailored approach to implementation (e.g., time of day, games played, culturally relevant activities). The program is strengths-focused by embracing and incorporating the unique knowledges, experiences, and subsequent strengths of each community in which the program is offered. In addition to community-specific program delivery, YAHLs, some mentors, researchers, and community leads participate in national gatherings to build a national IYMP community and provide youth with opportunities to share their experiences with each other. 

The IYMP has been shown to impact health-related outcomes, including reducing Indigenous children’s waist circumference and body mass index, as well as increasing nutrition knowledge and dietary self-efficacy [21]. In addition to these quantifiable health outcomes, exploring lived experiences may further our knowledge about perceived impacts of the program. Indeed, there is a well-established call for increased engagement with children and youth in research by honoring their experiences and insights [11]. Considering this, the best source of information relating to lived experiences in the IYMP would be those involved in the program. As the IYMP involves interconnected layers of mentorship and involves the active engagement of individuals in various roles, it is important to include YAHLs, mentors, and elementary students’ perspectives when exploring program benefits. 

Delivering culturally safe programming in an urban setting is complex as youth from various Indigenous groups and belief systems are engaging with non-Indigenous children. Therefore, the experiences and delivery will differ tremendously from what would be delivered within a single, rural/remote First Nation community, where the local beliefs and cultural practices are likely more uniform. The purpose of this study was to explore YAHL, mentor, and elementary student experiences in an *urban* offering of the IYMP. Research questions were twofold: (1) what are the experiences of individuals in various roles in the IYMP? (2) What are the impacts of the program, as perceived by individuals in various roles in the IYMP? Applying qualitative methods to explore our research questions helps address the calls to further investigate the effectiveness of Indigenous youth-focused health-promotion programs [13] and honor youth voices [11].

## 2. Methods

### 2.1. Strategy of Inquiry, Procedure, and Participants

The IYMP offering associated with the current study took place in an urban core neighborhood high school located in the Canadian prairies. Two groups of young people acted as program leaders and mentors: (1) YAHLs were Indigenous university students; and (2) high school student mentors were enrolled in a required grade 10 wellness girls class. The program took place over two school years and consisted of 45-min sessions during instructional time, one or two days per week for a total of 20 sessions per year. The program’s YAHLs and mentors spent instructional time on an additional day each week to plan that week’s session(s), and subsequently delivered the program to elementary school students from a nearby core neighborhood school. The YAHLs had attended multiple workshops, including national research gatherings, to learn about the Circle of Courage [19] and Four Rs [20]. Mentors were introduced to the frameworks at the start of each school year, in an attempt to root subsequent program planning in Indigenous values. Moreover, as part of program preparation, YAHLs and mentors were provided guidance on program planning and delivery (e.g., increasing activity time by not playing elimination games, the importance of learning participants’ names). Some examples of physical activities included in the program sessions were traditional Indigenous games (led by an Indigenous Knowledge Keeper), relays, and hoop dancing. Examples of the cultural teachings included storytelling during traditional games, a Cree language memory game, and Métis sash weaving. Examples of the healthy snack included pemican, fruit smoothies, and slices of fruit and vegetables. As an urban offering, this IYMP provided a novel approach whereby Indigenous and non-Indigenous peoples came together in an Indigenous-themed program. Indigenous student enrolment during the program was approximately 74% and 42% at the elementary school and high school, respectively. 

In an attempt to develop a comprehensive description of experiences in this urban IYMP offering, qualitative description was selected as the strategy of inquiry [22]. Qualitative description permits detailed and nuanced interpretation, which makes it ideal for exploring experiences of individuals in various roles in the IYMP. This research study represents a partnership between study investigators, local school division stakeholders, and Indigenous and non-Indigenous peoples involved with the urban IYMP offering. All of the YAHLs, mentors, and elementary students involved in this program offering had the opportunity to voluntarily participate in the study connected with the program. Elementary students, mentors, YAHLs, and guardians (where applicable) were provided with a pamphlet of information about the research component associated with the program. A community meal was also offered for the elementary students and their guardians to learn more about the study. Those who were interested consented and/or assented (and where needed, parental consent was provided) to study participation.

Through ongoing consultation with participating school division stakeholders (e.g., teachers, principals, school counselors, division coordinator), sharing circles were identified as a respectful, ethical, and appropriate way to explore IYMP experiences. Sharing circles provide opportunities to share experiences, stories, or views in a meaningful, respectful, and orderly way [23,24] and they encourage interconnectedness, dialogue, and co-creation of knowledge [25]. Sitting in a circle, which is symbolic of balance [26], individuals are asked one at a time to respond to a designated question or topic, with the option of not initially responding and instead listening to the views of others and contributing later in the discussion during progressive turns around the circle. Only one person speaks at a time, with that person’s turn indicated by holding a talking stick. The discussion ends when every individual believes there is nothing more to be added and talking has ceased. Data generation was led by three members of the research team, and a school division staff member passively participated (i.e., observed) in the sharing circles with the elementary students.

A total of 11 sharing circles were conducted; six with the elementary students (*n* = 23; 9–11 years old; grades 4 and 5; 3–4 elementary students per sharing circle), two with the mentors (*n* = 3; 15–16 years old; enrolled in a required grade 10 wellness girls class; 1–2 mentors per sharing circle), and three with the YAHLs (*n* = 6; undergraduate university students; 1–3 YAHLs per sharing circle). Every attempt was made to engage participants in sharing circles. There were two instances where participants were unable to attend a sharing circle and instead one-on-one interviews were conducted (i.e., one mentor interview and one YAHL interview. Sharing circle topics centered on recalled experiences in the IYMP (e.g., “What are some of your memories about the program?“), feelings about the program (e.g., “How did the program make you feel?“), perceived role in the program (e.g., “What did *you* do in the program?”), and lessons learned (e.g., “What have you learned?“). Sharing circle guides were developed by the research team in consultation with school division stakeholders. The primary differences between the elementary student, mentor, and YAHL sharing circles were the ways in which the topics and questions were addressed; for instance, elementary students were asked about their involvement as participants and learners in the program, whereas YAHLS were asked from their perspective as facilitators of the program. As the program was offered during school hours, we also held two focus group discussions with the school teachers and principals. Though these discussions were more logistical in nature (e.g., scheduling adaptations, managing off-task behaviors of mentors and elementary students), they provided further context and perspectives about the program. 

### 2.2. Data Analysis

Data analysis stemmed from a social constructivism interpretive framework [27], which recognizes that researchers’ backgrounds shape interpretations of the data. For instance, the analysis team was very familiar with the Indigenous frameworks guiding the program and recognizes that this familiarity likely shaped their interpretive lens. Further, the analysis team included Indigenous and non-Indigenous peoples, and individuals with varying years of qualitative research experience and years working with children and youth in a school setting (e.g., 2 years to over 25 years). Through a social constructivism lens, the analysis team acknowledges that social identities, backgrounds, and personal experiences cannot be removed from the data analysis process; rather, they are recognized as shaping data interpretation.

Data were analyzed through inductive content analysis, with one member of the research team leading the data analysis process. Following Elo and Kyngäs recommendations, content analysis consisted of three stages: preparation, organization, and reporting [28]. In the preparation stage, audio-recorded data were transcribed verbatim and multiple initial readings of all transcripts permitted familiarization with the data while reflecting on the meaning of the dataset as a whole [29]. More specifically, we treated the transcribed data as a collective in which all sharing circles and focus group discussions were analyzed as a whole, rather than separately analyzing by role in the program. In the organization stage, the data were re-read multiple times while documenting impressions, thoughts, and ideas. Open coding took place as sections of the transcripts were coded (meaningfully labeled) and eventually clustered into categories and content-specific themes. Three members of the research team engaged in multiple rounds of peer debriefing [30] during the data analysis process. These research team members served as “critical friends” [31] who enriched data analysis by encouraging a reflection of alternative explanations and interpretations. For example, the critical friends provided feedback on open coding (e.g., consideration for alternative labels), questioned code clusters (e.g., why or how do these codes cluster together versus a different cluster?), and posed alternative content-specific theme formations (e.g., consideration for differing ways themes might be formed). The reporting phase includes the presentation of themes and direct quotes from participants, with accompanying participant-selected pseudonyms. 

## 3. Findings

The IYMP offered a new experience for most of the elementary students, mentors, and YAHLs, and overall the program was received positively. The mentors and elementary students were excited to be involved, and they expressed feelings of enjoyment fostered by a fun and supportive environment: “(It’s fun) because you get to see everybody, you get to learn more, and you get to play new games.” (Patrice, elementary student). These positive views of the program were reflected by the majority of participants’ desires to participate more often. For example, Jocelyn (elementary student) expressed: “I wish that we could go all the time and not just some days because I really liked the program.” Beyond these positive sentiments about the program, three themes were developed that embody the experiences of those involved: *Fostering Wellness*, *Strengthening Meaningful Connections*, and *Exploring Leadership* (see Table 1 for a summary).

### 3.1. Fostering Wellness

The strengths-focused nature of the IYMP impacted the elementary students’, mentors’, and YAHLs’ wellness in various ways. Reported program benefits have been categorized into *physical wellness, emotional wellness,* and *mental wellness*; however, the lines between these categories were often blurred. For example, many participants explained that emotional and mental benefits were highly related to the physical activities they were engaging in during the program, which exemplifies that each wellness aspect is connected to and influences the others. 

#### 3.1.1. Physical Wellness

Participants enjoyed the two core IYMP components focused on physical health: healthy snacks and physical activity. The mentors were encouraged to think critically about the nutritional value of the snacks they were choosing, and subsequently, the elementary students were exposed to a variety of healthy options. Some elementary students shared that they had taken home recipes of certain snacks to make the snack at home. Similarly, the overall enjoyment of physical activity during the IYMP, as well as learning new ways to be active, translated to several elementary students indicating that they increased their physical activity outside of the program: “*I’ve been playing at the park more often and going swimming outside more often,*” (It’s Me, elementary student). Sir Blargon (elementary student) shared: “*I started…getting outside more. Ever since IYMP, I’ve been like going outside, taking a ride on my scooter, playing tennis with my friends.*” 

The IYMP provided opportunities to be physically active, which in turn included taking a break from school-related stressors:

“*With being a [university] student you just sometimes, you get so busy in life you kind of forget to... you don’t have much time to work out and stuff so I was glad to run around and participate in some of those games. I mean, it was to get movement for me, so it really helped me physically in that aspect.*”

(Batman, YAHL)

The YAHLs and mentors benefitted from engaging in *fun* activities that challenged their perspectives of why they engage in physical activity themselves. One of the YAHLs explained:

“*…it was nice because it was different from the activity I would typically be doing and kind of made you realize that physical activity isn’t just all working out per se; it can also be playing and just, you don’t even really realize you’re being–you’re exercising really because you’re just having fun. Um, so definitely made me kind of look at being active, I guess, in a new way or developing new ideas of how to be active.*”

(Harry Potter)

The elementary students’ perspectives of physical wellness were also expanding, as many of them expressed that learning *new* games/activities and learning about healthy eating helped them change some of their habits. Overall, the focus on physical activity–and subsequently physical wellness–in the IYMP translated into opportunities for mental wellness (e.g., learning new activities), exemplifying the ties between different aspects of wellness.

A focus of the physical activities included in the program (e.g., capture the flag, lacrosse, double ball) was cooperation and teamwork, which created a relational element to the physical component of the IYMP, as noted by Wonder Woman (elementary student) in Table 1. Indeed, physical activity was viewed as enjoyable by many participants because of the socio-emotional connections that developed while being active. Madeline, a mentor, explained:

“*I feel like for sure my physical, mental, and emotional aspects were like extremely attached with being able to have this [program]…I actually wanted to participate in because I’m not a big physical activity fan but when I’m leading with other kids, I’m kind of, like, not forced to be, like, you know, excited about it, but we have to be and so, um, that encouraged me to actually start doing it. So I was definitely, um, being more physically active.*”

Wonder Woman’s and Madeline’s sentiments exemplify the interconnected nature of wellness, and that the physical activities impacted perspectives of physical wellness in a positive and relational way. These experiences suggest that the program went beyond a sole focus on *personal* physical wellness by developing a *community* of active people who encouraged and supported each other to make physical activity fun and engaging. 

#### 3.1.2. Emotional Wellness

The perceived impact of the IYMP on emotional wellness was largely a result of the relationships that were developed with one another. Relationships generated in the IYMP provided safety, support, and comfort for many participants:

“*Going to the [university], I’m kind of out of my comfort zone, like in terms of being away from home and being away from my like support system…it was a tough semester for me this past year and I’m glad I made it through and just being a part of this program here really, uh, helped me emotionally…*”

(Batman, YAHL)

Common amongst all mentors and YAHLs was the feeling of contributing to something important and having a positive impact on others. This sense of pride is a notable strength of the program. Yuzuru (mentor) described how this connected to their emotional wellness:

“*When it’s positive growth that we’re trying to introduce into their lives and for us to be able to participate in this, that meant a lot to me because maybe in the future it might affect these children a lot more or maybe it’ll affect them less but in some way, shape, or form I’ve come to realize that it has affected me too in a positive way because whenever I saw the kids it was—it made my day better and I was much happier because I’ve realized that, you know, it’s possible that I might have been able to make, like, a small positive change in the world.*”

Batman (YAHL) also shared how IYMP held emotional meaning for them as a leader in the IYMP:

“*…like being involved with stuff like that, like volunteer-type extra-curricular stuff, it really, you know, makes me emotionally—it really helps me a lot knowing that you’re, you know, working towards a good goal and something positive and that you’re impacting people’s lives. I mean, that really, really made me feel really good.*”

While the leaders were experiencing emotional wellness because they had an important purpose in supporting the elementary students, the elementary students were also experiencing emotional wellness because of the support they were receiving, displaying reciprocity within the program. Some elementary students explained that they were able to regulate their emotions better as a result of their IYMP experiences. It’s Me (elementary student) shared: “*Yeah, um, I tried to really build my confidence because we were doing more activities. I feel like braver now because I can control more of my emotions.*”

The most common emotion mentioned when discussing the program was happiness. Jack (elementary student) shared how the opportunity to be present in a positive and happy environment impacted them (see Table 1). Jocelyn (elementary student) explained how happiness was, again, linked to relationships with other people in the program and a sense of belonging and acceptance: “*When I was at the program I was really, really happy because I met a lot of new people.*” Harry Potter (YAHL) added, “*That’s what the program is all about, is having fun and building relationships.*” 

The prominence of happiness was strong evidence that the program impacted participants’ emotional wellness.

#### 3.1.3. Mental Wellness

The perceived impacts of the IYMP on emotional and mental wellness were closely connected, making it difficult at times to clearly distinguish whether emotional or mental wellness best represented participants’ experiences. As a guide, the focus on mental wellness leans toward the mind, learning (including self-learning), and developing. Involvement in the IYMP helped some participants to build positive self-image and provided opportunities for growth: “*It helped me not be lonely and just not be quiet all the time and collect my words right instead of being shy all the time.*” (Amelia, elementary student). Connecting with one’s identity while engaging with others was an important aspect of some of the participants’ experiences, and reinforced the strengths-focused nature of the program. Participants expressed the IYMP was a safe environment to be themselves while also exploring new challenges:

“*I made a bunch of friends like you guys…helped me to not be afraid and just let me be myself there so I obviously made more friends and I learned how to be more, more gentle and more creative with myself than rather just copy other people.*”

(Josh, elementary student)

There were many examples shared of IYMP helping to reduce stress, which speaks to a positive attitude generated through the program. Both Alex (YAHL; see Table 1) and Elsa (elementary student) spoke about the calming effect of the program: “*Sometimes it would calm me down and help me not be nervous. It would just calm me down.*” Related to this is the stress-reducing effect that the program had on the YAHLs:

“*[I]YMP has always been a really good stress reliever for me and a really nice break in my day where you can kind of just let everything go and just play for an hour, so mentally and emotionally, um, it’s always helped—helped me kind of just let everything go and just have fun, um, be active for an hour which has been really great.*”

(Harry Potter, YAHL)

The IYMP also provided a variety of new learning opportunities for participants and this fostered an openness to further exploring unfamiliar activities, teachings, and ideas. Sarah (YAHL) explained, “*I think some of the things that we learned were really cool and I would like to share them and it did make me more interested in it.*” This motivation to learn and grow suggests that participating in the program impacted mental wellness.

### 3.2. Strengthening Meaningful Connections

The IYMP’s power of connectivity was discussed by all participants. The program facilitated connections to people, to culture, and to community and, at times, the connections operated in multiple ways. For instance, it was at least partially through building relationships that program participants engaged with culture, and building a connection with community included connecting with people. In an attempt to honor each connection, this theme looks at building relationships, growing culture, and lifting community.

#### 3.2.1. Building Relationships

Relationships were at the heart of the program and integral to its success:

“*I think one of the biggest positives and one of the biggest things that everyone got out of the program, um, is probably building those relationships with everyone and I think that was such a key part of the program.*”

(Harry Potter, YAHL)

The program facilitated the development of new relationships, as well as the growth of existing relationships. For the elementary students, engaging with people who were older than them was often mentioned as a favorite part of the program: “What I really liked is getting to meet everybody and making new friends” (It’s Me, elementary student). These new relationships held meaning for many of the mentors as well, as exemplified by Yuzuru (mentor) who enjoyed the chance to strengthen relationships with the elementary students (see Table 1). Multiple mentors agreed that building relationships contributed to the success of the program by allowing them to understand the elementary students’ interests and personalities, which they used to develop future sessions.

Integral to quality relationships within the IYMP was reciprocated respect; when individuals in one role offered respect to others, they received respect in return; and vice versa. There were instances when a lack of respect was evident; John Cena (elementary student) shared that sometimes the mentors “*would just get mad at us and yell at us.*” Though few in frequency, the effects of these instances included diminished feelings of belonging amongst the elementary students, while the mentors felt disrespected as leaders. 

When respect was evident, relationship-building created a sense of belonging. One elementary student stated, “*I felt like I was meant to be there*” (Elsa), and another elementary student explained, “*The more and more I went there the more I felt like I belonged there ‘cause it was really fun meeting all these people, playing games with them, and sharing stuff with them*” (Sir Blargon). Batman (YAHL) expressed their own sense of belonging:

“*I felt like I belonged with a lot of the First Nations kids that were there. Felt really good being there. The program was great, I mean the people you work with was really good…I think that’s key, you know, like having just team players in the program is really, really, really important.*”

There were times when relationship-building within the IYMP extended to impacting relationships outside of the program. Yuzuru (mentor) shared how the program motivated them to put more energy and time into important relationships:

“*I was able to kind of find more joy in hanging out with my siblings and being with them because usually I get along with them fine but you don’t necessarily go out of your way to just interact with them, so that was fun because I realized that some kids don’t really have older siblings to look up to or they—if they do, it’s kind of a tumultuous relationship, so I tried to reach out to them more.*”

This increased effort and appreciation of existing relationships external to the program speaks to the power of relationship-building within the IYMP.

#### 3.2.2. Growing Culture

As a core component of the IYMP, the YAHLs and mentors planned a range of cultural teachings (e.g., participating in traditional Indigenous games, teachings from Traditional Knowledge Keepers) that were memorable for many program participants. The increased awareness and knowledge of Indigenous cultures sparked a desire in some participants to continue learning: “*I’m definitely more curious and if I get a chance, I want to learn more,*” (Daisey, mentor).

The structure and philosophy of the IYMP is centered in an Indigenous worldview, which includes an inherent shared responsibility of learning and belief that learning is relational. It was apparent that participants in all roles of the program were learning and growing together:

“*I think every session and every year I learn more and more. There’s always people that know stories that you’ve never heard before or know of cultural activities that you’ve never seen or engaged in before so, um, I definitely learned more I think.*”

(Harry Potter, YAHL)

Many program participants reported enhanced engagement with the cultural component of the IYMP because of the shared responsibility for learning. Yuzuru (mentor) explains:

“*I felt as if I was able to connect more with what we were learning. Not necessarily that I learned more but that we were exploring it on a different level—kind of having to understand it and then explain to the kids for them to be able to understand it and for us to explore these concepts together was very important.*”

The YAHLs experienced a sense of pride in being able to share pieces of their own culture: “*I was really happy and excited to be able to share some things about Métis culture and that made feel really proud and excited to share.*” (Harry Potter, YAHL). Similarly, Alex (YAHL) expressed:

“*Practicing making the Métis sashes at home, my family was helping with me [laughter], like they were really excited to hear about this program and I think—my dad is where the Métis ancestry comes in my family, so I think he was really excited that I was making these connections, so I’ve shared a lot with them and then friends and other people at school. I think it’s important to know that these programs are here and they’ve been really beneficial so I’ve been trying to share a lot about what we’ve been doing.*”

Engagement with one’s own culture was also evident for some participants, including Shrek (elementary student; see Table 1) and Rhino (YAHL) who shared, “*Especially having grown up without knowing much of my heritage—my First Nations heritage—it was nice to have, uh, be involved in a program that was about educating on it and learning some of the cultures*”. The IYMP was therefore meaningful for some participants because they had the serendipitous opportunity to engage with cultural teachings that related to aspects of their personal identity. 

#### 3.2.3. Lifting Community

The connections built between elementary students, mentors, and YAHLs created an IYMP community that promoted acceptance, comfort, inclusion, and safety. Many of the mentors were aware of their influence in the creation and lifting of community within the IYMP. For example, Madeline’s (mentor) quote in Table 1 identifies the potential power of the program contributing to a safe space for the elementary students.

Some program participants also felt stronger connections to the wider community outside of those involved in the IYMP. Madeline (mentor) expressed “*We got to, um, combine kind of our two schools, um, which I think really helps bring together the community.*” Sometimes guest instructors or visitors from the larger community were invited to lead program sessions, again, fostering community connection:

“*I also liked the days where we brought in people from the community. Like the days that we did Pow Fit and then the Cross Fit days. I think that was really cool to bring in other people that had knowledge that we didn’t necessarily have and then we all got to do it together.*”

(Harry Potter, YAHL)

Elsa (elementary student) shared how nurturing community connections through the IYMP impacted their own feeling of safety while they were out in the surrounding neighbourhood: “*It made me feel safer because I love some of these places and I can walk to them*.” As a result of building and nurturing connections to the wider community, some mentors and YAHLs placed more value on community and were inspired to give back: “*It made me, I think, want to become more involved in volunteering activities in the community so I’m getting involved with those things too*.” (Yuzuru, mentor). This suggests a potential rippling effect of lifting community that was fostered in the IYMP.

### 3.3. Exploring Leadership

Many of the mentors and YAHLs viewed a large part of their responsibilities as being a leader and role model for the elementary students, and they valued this important role: “*I think that was my favourite thing, is like seeing the way they look up at us and like how much they actually value like, what we’re teaching them*.” (Madeline, mentor). Batman (YAHL) shared:

“*Any time that I can be present in a school setting like that is really important so I know to be a good role model and to, you know, let kids know that, uh, especially First Nations, let them know that us too, we can flourish in those environments and, you know, in those settings, you know, be active and be able to participate in extra-curricular activities and sports, especially too.*”

Mentors and YAHLs displayed leadership not only by leading group activities, but also by leading by example and finding ways to engage with students who required more support and encouragement. As touched on by Daisey (mentor), they had the opportunity to practice and build a variety of skills and qualities related to leadership, including patience, communication, responsibility, initiative, and time management, amongst others (see Table 1).

These leadership qualities were required from the mentors to create a positive experience for the elementary students. If ever leadership was lacking from the mentors, who themselves were still growing and learning, it negatively impacted the elementary students’ enjoyment of the program. 

Embracing a leadership role required many individuals to step outside their comfort zone. Given the high school students were serving as mentors as part of a high school class, as opposed to volunteering to be a mentor, this presented challenges for some who were “*…uncomfortable and they retracted*” (Ashley, teacher). For others, they immediately embraced working with the elementary students and rose to the position of leader:

“*By having it like as a course requirement to take part in this program, um, I think we got girls engaged with children and working with children and seeing strengths in themselves that we—they wouldn’t have been able to find had this program not existed.*”

(Ashley, teacher)

Many of the YAHLs and mentors embraced their leadership role and looked for ways to grow in their roles. Alex (YAHL) explained, *“…I’m quite shy, so I actually think this program really helped me, um, get out of my shell a little bit, so I think I gained a lot of, um, confidence in being in more of a leadership role*.”

The willingness to bravely engage in new experiences resulted in enormous growth:

“*Our role was to grow ourselves, to grow as our own people and become more accustomed to interacting with others, especially younger children, in being able to communicate well and participate in these activities and learning these leadership skills and community values.*”

(Yuzuru, mentor)

Madeline (mentor) offered another perspective about growing as a leader:

“*…for us it was like a learning experience, you know? Like learning how to lead in our communities and stuff and realizing how easy it is to, like, make an impact. And then our role was also to be, you know, show these positive characteristics and stuff and so that the [elementary students], who look up to us, can like see those, um, and learn and be like, ‘That’s what like, you know, a good high school student is like.’ It’s like, ‘That’s who I want to be,’ kind of thing and so that they can take that and incorporate that into their own lives I think and just, like, be that role model for them.*”

The IYMP provided the opportunity to practice leadership skills in a safe context. One mentor shared that this experience has affected and reaffirmed their future career goals: “*When I’m older I want to be a teacher and so this gave me a really good perspective on, like, what that’s going to be like I think,*” (Madeline). The IYMP may have a lasting impact on the opportunities that Madeline and other mentors decide to pursue in the future.

#### Mentorship

Both the mentors and YAHLs recognized the importance and impact of mentorship and building relationships as part of their leadership roles. Though mentorship may not always be considered an inherent part of leadership, being a mentor was identified as a salient component of leadership experiences in the IYMP.

“*[In the first few sessions] we were unable to communicate clearly because we didn’t really have an understanding in how to interact with younger children, even us with younger siblings because it’s different when you’re interacting with kind of complete strangers and you try to become closer with them, like an older sibling figure, so later on in during later dates, when we were doing IYMP, it was easier for us to speak with them and to get them to do the activity that we were planning, to get them to participate.*”

(Yuzuru, mentor)

Yuzuru speaks to the positive impact of the ongoing mentorship relationships with the elementary students and how this affected program implementation. Rhino spoke to how they felt in the mentorship role as a YAHL, and hopes for both a good and lasting impression as a role model (see Table 1).

An inherent feature of the IYMP was that each role in the program was supported by other roles, and this was recognized by many participants. The elementary students recalled that they had people to turn to when they needed help. Brenda Bob (elementary student) shared that they “*liked the [mentors] ‘cause they helped us out,” and Amelia (elementary student) took comfort in knowing that “there is always someone there for you if you need it*.” Support also flowed from the YAHLs to the mentors. Harry Potter (YAHL) explained that their role was “*… guiding them [mentors] along and um, encouraging them to come up with their own ideas and encouraging them to participate.*” Alex (YAHL) expanded:

“*The students that weren’t participating or kind of hesitating, like, I tried to really talk with them and get them to be more engaged because I think this was about bringing everyone together, so some of the quieter students that didn’t want to help out—the high school students at first—tried to support them and make them feel welcome and that they can step up and be that mentor as well. I think, um, was a role that we had as university students that I think was beneficial.*”

This helping hand to those that needed it was very important to fostering relationships between YAHLs and mentors with elementary students.

Something that hindered the impact of mentorship within the program was the lack of young men as mentors. As a girls’ high school class served as the group of mentors, the elementary boys lacked men role models/mentors, which impacted experiences in the program:

“*That was one thing that the [elementary students] said that—they didn’t like that they didn’t have kind of a group of guys. The guys felt like, ‘I’m not gonna go up to a girl and ask if she wants to be my partner.’ So…they felt really out of place.*”

(Shopkins, teacher)

There were a few men YAHLs involved in the program that, when present, facilitated an increased sense of belonging, comfort, fun, and support for the elementary boys. However, the attendance of these men YAHLs was sporadic due to other commitments, creating a challenge in trying to reach *every* student through mentorship.

## 4. Discussion

We qualitatively explored YAHL, mentor, and elementary student experiences in the IYMP, and in doing so we identified a number of perceived impacts of the program; namely, wellness, connectivity, and development of leadership skills. Rather than the success of the program and potential impacts falling on the responsibility of a select few, every role in the IYMP contributed to fostering wellness, connections, and leadership. While the findings of our qualitative description stood near to the data, we offer nuanced interpretation [32] by elaborating on three points: (1) mentorship, (2) wellness, and (3) the urban context.

Mentorship is an inherent feature of the IYMP [33], and our results indicated that mentorship was experienced by individuals occupying various roles. Beyond the individuals involved in the IYMP offering associated with this study, the national IYMP team consists of youth, Elders, community leaders, teachers, principals, trainees, project personnel, and researchers from across Canada. As part of the larger IYMP team, individuals, regardless of their role (directly or indirectly associated with a program offering) have a range of connections, supports, and resources available to them through other members of the national team. Essentially, mentorship prospers within the larger IYMP team. Our interpretation of mentorship within the IYMP (both the urban offering associated with this study and the larger IYMP team) supports the non-hierarchical mentorship model proposed by Halas et al. [33] in which mentorship is circular rather than hierarchical. This approach to mentorship is not uni-directional [17]; rather, each individual shares their gifts and respects one another enough to recognize that others will share theirs. The results of the current study suggest that this IYMP offering included multi-directional mentorship whereby individuals in various roles contributed to the program and offered their strengths to others. Given the perceived benefits from this IYMP offering, other Indigenous youth-focused programs might consider adopting a circular and multi-directional, or communal-led, mentorship approach.

Unanticipated mentorship may have also contributed to perceived program impacts. The IYMP offering described in this paper included unintended mentorship beyond that from the YAHLs, mentors, and national IYMP team. For instance, having the program delivered during school hours as part of students’ classes meant the involvement of teachers and principals. Though their novel inclusion led to some uncertainty regarding their roles (e.g., what should teachers be doing when a communal-led mentorship program is part of their class?), the involvement of these individuals provided unexpected mentorship opportunities. For instance, teachers offered gentle suggestions to YAHLs and mentors on how to get students engaged. As another example, as a member of the research team our community advisor provided invaluable guidance within the school system and facilitation with school staff and school leadership. These examples of unanticipated mentorship are a form of partnership development between researchers, practitioners, and various knowledge users. Our research, therefore, contributes to a larger body of literature that underscores the importance of developing meaningful partnerships to enhance program development and implementation [10]. For instance, researchers and program planners might formalize a community of practice [34] or adopt an implementation science framework (e.g., Knowledge to Action Framework [35]) to guide a dynamic process of decisions and actions pertaining to program development and program evaluation.

As a healthy lifestyle program, it was perhaps not surprising that participants identified positive impacts on their wellness, namely physical, emotional, and mental dimensions of wellness. Wholistic wellness, consisting of physical, emotional, mental, and spiritual wellness, is essential for human potential [36,37]. Absent from the theme *Fostering Wellness* in the current study was spiritual wellness. A few of the YAHLs spoke of the personal engagement they experienced between learning Indigenous cultural teachings and their own spirituality. However, spiritual wellness was discussed to a much lesser degree and from fewer participants than other wellness dimensions. Given that all of the YAHLs self-declared their Indigenous identity, some components of the program may have more closely aligned with their own spiritual wellness than non-Indigenous mentors and elementary students. Spiritual wellness has been affirmed as an important aspect of maintaining *human* wellness, not just Indigenous peoples’ wellness [38]. It is possible that spiritual wellness may have been impacted, but it is often identified as “difficult to explain” [39]. Personal and cultural variations in the definitions, meanings, and experiences of spirituality [38] may have further added to a lack of direct discussion on this aspect of wellness.

Although not dimensions of wholistic wellness, autonomy and self-determination are meaningful for health and wellness. Autonomy is an innate psychological need referring to the desire to self-organize experience and behavior in a way that aligns with one’s sense of self [40]. Self-determination is a determinant of Indigenous peoples’ health [6], and programs that are rooted in the needs and priorities of communities can support wellness [41]. In many ways, the IYMP is premised on self-achieved wellness. The program is based on communal mentoring in which mentors (with YAHLs’ supervision and facilitation) develop and deliver a program based on their own preferences and strengths, as well as their community’s needs or interests. The urban IYMP offering presented in this study took place during school hours and was part of the mentors’ and elementary students’ classes. Although the program was built on YAHL and mentor abilities, talents, and energies, there may have been less autonomy in this prescribed mentorship program versus a program where individuals elect to be mentors (i.e., outside of class time). Given the wellness benefits associated with autonomy, future class-based offerings of the IYMP may need to explore intentional ways to heighten autonomy and create intrinsically motivated mentorship.

It is important to note the unequal gender representation among leaders in the program. The school division associated with this program has separate grade 10 wellness classes for boys and girls, and school division partners had selected the girls’ class to be mentors for the program. Moreover, there were nearly double the number of women YAHLs compared to men YAHLs across the two years the program was offered. This gender disproportion in the program is echoed in the literature where, for example, Indigenous women surpass Indigenous men in educational attainment [42]. Having more women YAHLs may have facilitated relationship building and connectivity with the mentors (and elementary students) in the program, who saw healthy, strong Indigenous women in a leadership role. However, mentors and elementary students, in particular the young boys, missed the opportunity to connect with more Indigenous young men as leaders in the program. The low number of male leaders may have impacted the planning and delivery of the program (and subsequently, experiences in and perceived outcomes of the program), as it is unknown the direction the program would have taken with more equal gender representation. It may be fruitful to work with community partners to intentionally support more Indigenous young men as program leaders.

The IYMP offering associated with this study was unique in that it was one of only a few urban-based IYMP programs (see [33] for program evolution). At the time of this research, the vast majority of IYMP offerings took place in rural or remote Indigenous communities. Given the urban context, and that the program was not located in a specific Indigenous community, the *Growing Culture* sub-theme is particularly meaningful because establishing cultural relevance can be challenging in an urban community research setting [10]. The program appeared to respectfully and appropriately incorporate cultural practices (e.g., including Traditional Knowledge Keepers), and program participants seemed to adopt a position of respectful curiosity to learn about various Indigenous cultural teachings and activities. This was evident in the YAHLs and mentors’ planning of program sessions that incorporated a range of cultural teachings and activities (e.g., Cree language activities, Métis sash weaving, hoop dancing, jigging), as well as the elementary students’ excitement to participate in those activities. Having a program that was rooted in the Circle of Courage [19] and worked to actualize the Four Rs [20] likely nurtured this respectful curiosity. Moreover, the First Nations, Inuit, and Métis Education Unit within the partnering school division provided invaluable support and guidance regarding the respectful inclusion of Indigenous content in a manner that fostered partnerships and encouraged authentic community engagement. Supporting cultural relevance and cultural safety is important when creating health and activity programs in the context of Indigenous youth programming [10]. Based on the current study, future urban-based programs may seek to create cultural relevance and cultural safety through a strong foundation of Indigenous values, centering Indigenous peoples’ voices (e.g., Indigenous YAHLs), and embracing communal mentorship (e.g., working with an Indigenous education unit).

This urban offering was also unique in that it took place during school hours to meet the needs of the community, whereas almost all IYMP offerings across Canada were afterschool programs at the time. To accommodate class schedules of both the high school and elementary school, the typical 90-min IYMP sessions were shortened to 45 min, which required flexibility and creativity to integrate core program components in a reduced time (e.g., eat a healthy snack while participating in a sharing circle, receive a cultural teaching while participating in traditional games). As the program took place during class time, teachers linked the program to various physical education, wellness, and health education curricular outcomes. It is important to recognize the unique context of this IYMP offering and the adaptations made by the community, as the contextual features may have contributed to perceived impacts of the program.

As an urban offering, the program included both Indigenous and non-Indigenous mentors and elementary students. The inclusion of non-Indigenous peoples in an Indigenous-based program provided an opportunity for non-Indigenous peoples to respectfully learn about Indigenous ways of knowing, being, and doing. The potential benefits of learning Indigenous ways of knowing, being, and doing include enriching, expanding, and challenging non-Indigenous approaches to wellness [43]. It has been proposed that non-Indigenous peoples may flourish when making space for and learning from Indigenous traditions and knowledges [43]. As presented in our findings, there was evidence of program participants appreciating the cultural teachings, and some who embraced the opportunity to learn more about their own culture through the program (e.g., Cree language, Saulteaux wellness wheel). Further, learning about diverse and unique Indigenous nations, tribes, and communities may help offset incorrect, inappropriate, and unethical pan-Indigenous attitudes and beliefs [44,45]. This program may, therefore, be considered a form of reconciliation between Indigenous and non-Indigenous peoples in Canada by supporting Indigenous cultural relevance in an urban-based wellness program [10].

### 4.1. Strengths and Limitations

We embrace a relativistic approach and invite the reader to adjudicate our qualitative study based on its context, conditions, and purpose [46]. In doing so, we present characteristics of our research that may prompt consideration for its strengths and limitations. Purposeful sampling [22,47,48] was used to ensure study participants occupying various program roles could provide information about IYMP experiences. Our research included ongoing community consultation, with direction and guidance continually received from our community advisor in particular. The community advisor contributed to our research in a variety of ways: linking researchers with the school division’s First Nations, Inuit, and Métis Education Unit, facilitating communication with school division staff, peer debriefing during data analysis, contributing to knowledge translation activities, and engaging with the national IYMP team. Our findings include examples (e.g., direct quotes) that countered main findings as a way to highlight opposing views or unique perspectives that cannot easily be categorized [30]. Prior to knowledge translation, the study was reviewed by the IYMP team’s national advisory circle which includes youth, Elders, community leaders, trainees, and researchers from across Canada. This process is part of the IYMP team’s code of ethics, which is based on the Kahnawà:ke Schools Diabetes Prevention Project Code of Ethics [49].

### 4.2. Future Directions and Conclusion

Building on the findings reported in this study, three directions for future research are proposed: (1) exploring the potentially unique benefits emanating from programs with prescribed versus elected mentorship; (2) directly examining the benefits of learning about and experiencing Indigenous ways of knowing, being, and doing for non-Indigenous program participants; and (3) program evaluation that embraces a culturally responsive approach. As discussed elsewhere [10], it will be important for future researchers to embrace culturally relevant practices and develop partnerships to co-create relevant, respectful, and relational research practices. The current study found that YAHLs, mentors, and elementary students perceived an urban IYMP offering to result in wellness, connections, and leadership skills. Given that the program offering was part of the national IYMP team premised on multi-directional mentorship, a robust exploration of the larger team would provide a wealth of knowledge about program benefits beyond individual program offerings.

## Figures and Tables

**Table 1:** List of Themes and Sub-themes with Sample Quotes.

Theme	Sub-Theme	Sample Quote
Fostering Wellness		
	Physical Wellness	My favourite part of the program was that we were actually all united and being really active. (Wonder Woman, elementary student)
	Emotional Wellness	I actually forgot about most things that were bad in my life and thought about the positive stuff because when I went there…you feel happy and you’re welcome there. It’s not that you’re sad, so I calmed down. (Jack, elementary student)
	Mental Wellness	Sometimes, um, I was coming from work and felt really, like, hectic and rushed …but then once you get there and like, everything’s come together…I think it really was, um, beneficial for my mental health. (Alex, YAHL)
Strengthening Meaningful Connections		
	Building Relationships	I think, personally, my favourite and the best overall experience that we ever had is really getting to connect with the younger children and seeing us bridge these gaps of being somewhat awkward during the first few sessions… so that was very nice seeing the growth that we have with our relationships with the younger kids. (Yuzuru, mentor)
	Growing Culture	I think I’ve learned some more and got closer to my culture…‘cause of the cultural teachings. (Shrek, elementary student)
	Lifting Community	…it’s super important to, like, make connections and stuff because, um, especially, you know, you don’t know what these kids in these communities are going through, um, and it could be, like, their safe place and I just feel like your goal is just try to make them, like, feel as welcome as possible. (Madeline, mentor)
Exploring Leadership		You are the older one, so you have to be the responsible one, the respectful one, the patient one especially, so I’d say if you have a positive attitude, it’s going to work out and you’ll have fun and learn new things. (Daisey, mentor)
	Mentorship	They look up to you, they tend to by the time you’re done, they have a lot of respect for you and you know, sort of become like a hero towards them. You just sort of hope that the impression you left with them lasts and it was good one in the first place. (Rhino, YAHL)

*Note.* YAHL = young adult health leader.

## Data Availability

Not applicable.
